# Surgical treatment of children with total colonic aganglionosis: functional and metabolic long-term outcome

**DOI:** 10.1186/s12893-018-0383-6

**Published:** 2018-08-15

**Authors:** Cristian Urla, Justus Lieber, Florian Obermayr, Andreas Busch, Roland Schweizer, Steven W. Warmann, Hans-Joachim Kirschner, Jörg Fuchs

**Affiliations:** 1 Department of Pediatric Surgery and Pediatric Urology, University Children’s Hospital of Tuebingen, Hoppe-Seyler-Str. 3, 72076 Tübingen, Germany; 2 Department of Pediatric Surgery, University Hospital Marburg, Baldingerstraße, 35043 Marburg, Germany; 3 Pediatric Gastroenterology, Department of Pediatrics, University Children’s Hospital of Tuebingen, Hoppe-Seyler-Str. 1, 72076 Tübingen, Germany; 4 Pediatric Endocrinology, Department of Pediatrics, University Children’s Hospital of Tuebingen, Hoppe-Seyler-Str.1, 72076 Tübingen, Germany

**Keywords:** Total colonic aganglionosis, Surgical treatment, Functional outcome, Metabolic outcome

## Abstract

**Background:**

Total colonic aganglionosis (TCA) is a rare variant of Hirschsprung’s disease occurring in 3–10% of the cases. Only few studies reported the long-term clinical and metabolic outcomes of patients with TCA. The aim of this study was to evaluate the functional and metabolic long-term outcomes of children undergoing surgical treatment for TCA.

**Methods:**

A 15-year retrospective study was performed. Blood chemistry tests and stool analysis performed at the last follow-up visit were recorded. Height and weight development were assessed using the corresponding percentiles for age. Faecal continence and quality of life were evaluated using a detailed questionnaire.

**Results:**

Eleven patients were included in the study. The median age at surgery was 6 months (range: 3–72 months). After histological confirmation, all patients underwent a total colectomy. Ileoanal anastomosis (*n* = 6), ileorectal anastomosis (*n* = 1), J-pouch (*n* = 1) and Duhamel procedure (*n* = 3) were performed. Temporary ileostomy was closed after a median of 8 weeks in 10/11 patients. After a median follow-up of 78 months (range: 27–199 months), all evaluated patients were continent. Height and weight were appropriate for age in only 5 patients. Vitamin B12 and folic acid serum levels were normal in all examined patients. Ten patients had normal hemoglobin serum levels. Seven patients had low transferrin saturation in serum. Hemoccult tests were negative in all examined patients. Despite complex postoperative courses in some cases, patients and parents showed good overall satisfaction in terms of quality of life.

**Conclusion:**

The majority of patients reported a good quality of life. This can result from the adaptation of the patients to certain disease states. The failure to thrive seems to be related with the extent of aganglionosis. The inclusion of these patients in interdisciplinary long-term follow-up care, in which pediatric surgeons, gastroenterologists, and dieticians are involved, is essential.

**Electronic supplementary material:**

The online version of this article (10.1186/s12893-018-0383-6) contains supplementary material, which is available to authorized users.

## Background

Total colonic aganglionosis (TCA), defined as aganglionosis extending from the anus up to 30–50 cm proximal to the ileocecal valve [1, 2] is a rare variant of Hirschsprung’s disease (HD), occurring in approximately 3–10% of all cases [3]. It represents a major challenge for both surgeons and pediatricians not only because it is difficult to diagnose but also because, once diagnosed, it may pose pre- and postoperative management challenges. Over the last several decades, greater awareness of this disease and improvements in the postoperative care have led to a dramatically lower mortality rate, which is now less than 5% [4]. However, morbidity remained significant [5].

The aim of surgical treatment is to remove the aganglionic intestine and to provide patients with a good quality of life, which is reflected in fecal continence, acceptable frequency of bowel movements, and no symptoms of enterocolitis [6]. Over the time, many surgical techniques have been described [7–10]. However, a meta-analysis from 2009 did not demonstrate superior results for any of them [11]. Nevertheless, all surgical techniques have several common characteristics, namely that negative consequences regarding nutrients absorption, continence, and quality of life may occur. Many patients are at risk for complications, including failure to thrive, anemia, electrolyte imbalances, dehydration, incontinence, and perianal dermatitis.

Only a few investigators have evaluated the long-term clinical outcomes and bowel functions of patients with TCA [4, 12, 13]. Even fewer reports have addressed the metabolic problems that may become apparent in these patients [1, 14].

This report details the functional and metabolic outcomes of patients after surgical TCA management who were treated at our institution.

## Methods

A retrospective data analysis of patients with TCA treated at our institution between May 2002 and September 2017 was carried out. The study was approved by the local ethical committee (number 470/2015R). The patient’s charts were reviewed for clinical presentation, diagnostic studies, number and type of operations, postoperative complications, and outcomes. Postoperative complications were categorized according to the classification system proposed by Dindo and Clavien (see the Additional file 1) [15].

As quite modest ileal resection in addition to colectomy may lead to rapid bowel transit and marked malabsorption of nutrients after surgery, all patients were regularly seen by our interdisciplinary team consisting of pediatric surgeons, gastroenterologists, and nutritional specialists. To this purpose blood chemistry tests (hemoglobin, iron, transferrin saturation, folic acid, and vitamin B12), stool analyses (pH, glucose, and hemoccult test), and a contrast study performed at the last follow-up visit were recorded. Growth and nutrition were assessed by recording height and weight at the time of the last visit; the corresponding percentiles by age for the population of our region were calculated and compared with those obtained at previous presentations.

Fecal continence was evaluated using a modification of the score proposed by Wildhaber [16] as shown in the Additional file 2. The parents were asked by phone to provide answers to a detailed questionnaire. The questions focused on the frequency of bowel movements, fecal soiling, stool consistency, and long-term use of medications (enemas, drugs). Scores ranging from 13 to 18 points indicated normal continence, scores between 7 and 12 points revealed fair continence, while those between 0 and 6 points revealed poor continence. Quality of life was assessed using the scoring system proposed by Barrena [12], as shown in the Additional file 3. The maximum possible score was 15 points. Patients with scores between 11 and 15 were considered to have good quality of life, while those having scores ranging from 6 to 10 and 0 to 5 were considered to have fair and poor quality of life, respectively. Poor outcome was defined as the presence of permanent stoma. The evaluation of continence and quality of life was only performed in patients ≥6 years of age. Descriptive statistics for continuous variables were expressed as medians and ranges.

## Results

### Study population

A total of 114 patients were diagnosed with HD at our institution between May 2002 and September 2017. Among these patients, 11 (9.6%) had TCA, including three females and eight males who had a median age of six months at diagnosis (range: 3–72 months). One patient had a family history for both HD (father) and TCA (younger sister). Associated malformations and syndromes were hemangioma of the scalp (*n* = 1), volvulus of the small intestine (*n* = 1), Mowat-Wislon syndrome (which includes vaginal duplex, pulmonary artery syndrome, microcephaly, growth and mental retardation, and epilepsy; *n* = 1 patient). Association of trisomy 21 and cardiac anomalies (bicuspid aortic valve, atrial septal defect) were encountered in two patients.

### Clinical presentation

The majority of patients (*n* = 7) were diagnosed with HD in the first three months of life. Among these 7 patients, only 2 patients were diagnosed with HD during the neonatal life (age at diagnostic: 4 weeks). The remaining 4 patients were diagnosed at four, nine, 14, and 30 months respectively. The most common presenting features, observed in nine patients, were abdominal distension associated with bilious vomiting and failure to pass meconium. The other two patients presented with toxic megacolon.

A barium enema was performed during the initial diagnostic workup in ten of the eleven cases. The radiological findings were nonspecific and only allowed for the recognition of a functional ileal obstruction. In only one case the radiological appearance was that of a microcolon. Full thickness rectal biopsy and histochemical studies for acetylcholinesterase were performed in all cases.

### Surgery undertaken before the definitive pull-through procedure

Nine patients required a decompressing ileostomy before the definitive operation. The ileostomies were placed at 15 cm from the ileocecal valve (authors’ standard practice). The median age at the time of placement of ileostomy was 33 days (3–420 days). In two of these nine patients, full-thickness colon biopsies were also taken because of the pathologic macroscopic aspect of the colon (thick walled, lack of peristalsis). The histological examination of these biopsies confirmed TCA. In the remaining patients, the extent of aganglionosis was established at the time of definitive surgery based on intraoperative frozen sections.

In the remaining 2/11 cases the ileostomy was placed at the time of definitive surgery. In these cases, ileal decompression was considered at the time as not necessary, as the patients could be managed conservatively using rectal enemas until the time point of pull-thorugh procedure.

Two cases worth special attention as they illustrate how the lack of specificity of the symptoms may pose difficulties in establishing the diagnosis. In the first case, it is about a female patient with trisomy 21 by whom an ileostomy was placed at 4 days of life due to meconium ileus. The preoperative contrast study revealed no caliber difference at the level of the colon. At this point, a rectum biopsy was not performed. Four months after surgery the ileostomy was closed. Two years after ileostomy closure the patient further developed obstructive symptoms which made necessary a laparotomy. Intraoperatively massive adhesions and stenosis at the level of the ileocecal valve as well as lack of peristalsis in the last 7 cm of the terminal ileum were found, so that a ileocecal resection was performed. Three months later the patient was re-admitted in our department due to recurrence of the obstructive symptoms. A rectum biopsy was performed, which confirmed the diagnosis of HD.

In the second case, it is about a male patient who was treated in another institution. He received on the 6th day of life a decompression ileostomy because of meconium ileus. Four months after ileostomy closure a re-laparotomy was performed due to adhesional ileus. Two months after this procedure the placement of an ileostomy was again necessary due to recurrence of the obstructive symptoms. At this point a rectum biopsy was performed and the diagnosis of HD confirmed.

### Definitive pull-through procedure

Definitive surgery was performed in all cases. The median age at the time of surgery was six months (range: 3–72 months). Total colectomy, endorectal pull-through, and straight ileoanal anastomosis was performed in six children. One patient received an end-to-end ileorectal anastomosis (Rehbein’s procedure). In three cases, a Duhamel procedure was carried out. In one case, a J-pouch ileoanal anastomosis was performed. This latter case underwent definitive surgery at another center but was referred to our institution because of J-pouch dysfunction.

The extent of aganglionosis was established at the time of definitive surgery in 9/11 cases. In the remaining 2/11 cases, the diagnosis of TCA was established before definitive surgery by colon biopsy, which were taken during surgery for placement of decompressive ileostomy as mentioned above.

The level of aganglionosis was limited to the colon in eight cases and involved the last 10 cm of the terminal ileum in the remaining three cases.

Intraoperatively, the colon appeared thick-walled and distended (*n* = 5 cases) or had the aspect of a microcolon (*n* = 2 cases). In the remaining 4 cases, the intraoperative findings were nonspecific.

### Postoperative course

To objectively exclude the presence of a postoperative stenosis of the ileoanal anastomosis, a rectal examination and calibration with a Hegar instrument under general anesthesia was performed in 10 patients after a median time of 4 weeks (range: 1–8 weeks). In the remaining patient, the parents refused examination because of an uneventful postoperative course. All examined children showed no evidence of stenosis. The closure of the ileostomy was carried out after a median of 8 weeks (range: 6–24 weeks). In one patient, the ileostomy was closed at the time of definitive surgery.

Details regarding postoperative complications are given in Table 1. No early postoperative complications were encountered. The most common complication was perianal dermatitis, which occurred in almost all patients and was most severe in the patient who underwent closure of the ileostomy at the time of definitive surgery. All patients were managed conservatively using zinc based ointments, and the problem consistently improved over time in all affected patients. One child developed an isolated stenosis at the site of the ileorectal anastomosis 14 months after surgery and required a Soave redo pull-through procedure. This patient suffered from Mowat-Wilson syndrome and had a further protracted course in the following period. She developed an ulcerative ileitis, which caused a severe bleeding anemia. Eventually, syndromic comorbidities led to the need for a permanent ileostomy.

**Table 1:** Postoperative complications

Complications^a^	n	Management	Grade^b^
Perianal dermatitis	9	Local treatment	I
Stenosis of anastomosis	1	Re-do pullthrough	IIIb
Obstructive ileus	2	Laparotomy, adhesiolysis	IIIb
Sphincter achalasia	2	Botulinum toxin injection	IIIb

^a^Median follow-up: 78 months (range: 27–199 months)

^b^Grading after classification of Dindo and Clavien [13]

Obstructive gastrointestinal symptoms such as stool retention during the day with elimination of explosive stools at night were observed in two patients after the operation at one and three years, respectively. The clinical examination under general anesthesia revealed a non-relaxing internal anal sphincter. In both patients, botulinum toxin injections of the internal anal sphincter were performed. In one patient, the symptomatology significantly improved after a single application. In the other patient, the symptoms recurred and repeated injections at intervals between three and five months were necessary (a total of seven injections were administered).

No pre- or post-operative deaths occurred. Moreover, none of our patients developed electrolyte disturbances or bouts of enterocolitis.

### Outcome and follow-up

The median follow-up interval was 78 months (range: 27–199 months).

Evaluation of fecal continence and patients’ and their parents’ quality of life was possible in seven cases (63%). The remaining four children were not suitable for evaluation because three were younger than six years of age, and one had a permanent stoma. After a median follow-up of 78 months (range: 27–199 months), the median frequency of bowel movements was reported to be 5/day (range: 4–8 stools/day). All evaluated patients were continent. Regarding the functional results of the surgery, the median modified Wildhaber score was 14.5 points (range: 8–16 points, total possible score: 18 points). Regarding quality of life, the median modified Barrena score was 14 points (range: 10–15 points, total possible score: 15 points).

Blood count and chemistry evaluation were obtained from eight children. In the remaining patients, the parents refused to allow the childrens’ examination. The majority of patients had a normal hemoglobin level, although four showed low transferrin saturation (< 10%). One patient presented with a hemoglobin level < 7 g/dl at his most recent visit and required repeated blood transfusions. Vitamin B12 and folic acid serum levels were normal in all examined patients, and showed no signs of vitamin malabsorption or bacterial overgrowth.

Stool analysis was also possible in eight patients. The majority (*n* = 6) had acidic stool (pH < 5.5). However, none of these patients presented with traces of glucose in their stools. The hemoccult test was negative in all of the examined patients. Only one patient showed a high level of stool calprotectin (1763 μg/g, normal value: 0 - 110 μg/g). A possible explanation for this high level may be that the examination was performed after an episode of gastroenteritis.

**Fig. 1:**
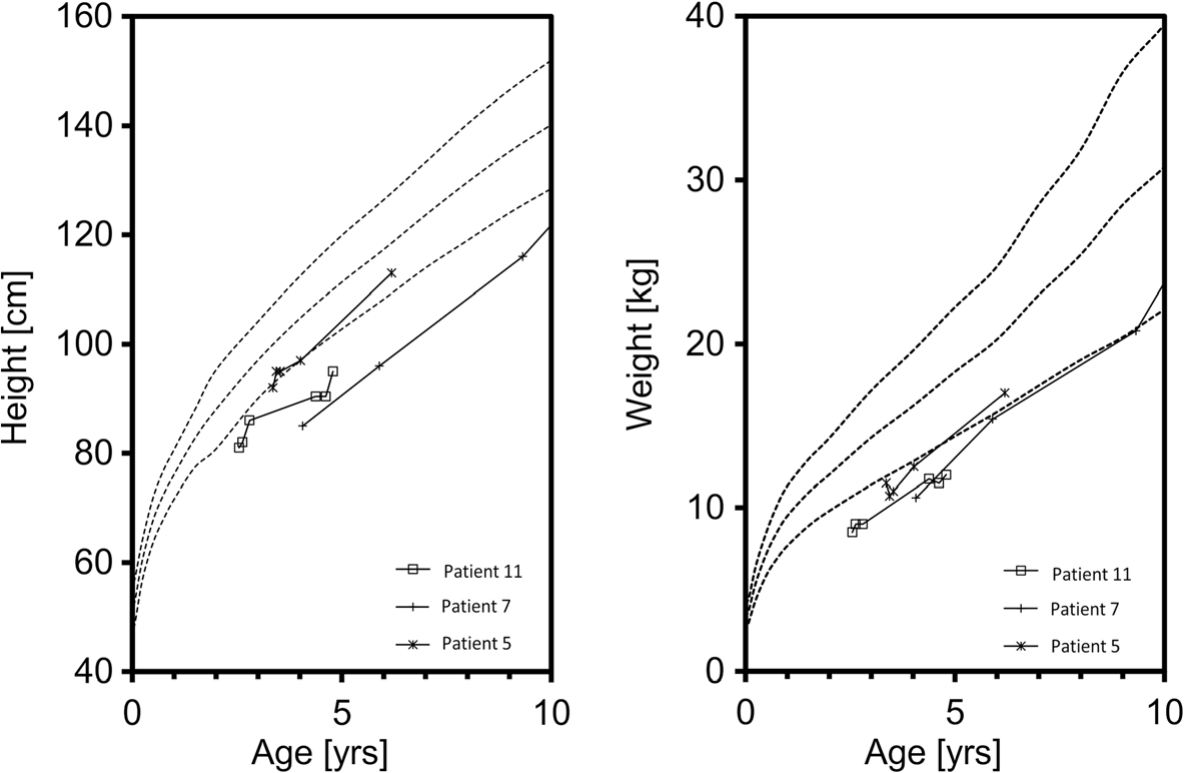
The 3rd, 50th, and 97th percentiles according to the height and weight references of Prader for female patients with a poor height and weight development. Patient 5 and 11 had inadequate height and weight gain and remained below the 3rd percentile. Patient 7 presented a borderline development parallel and slightly above the 3rd percentile

**Fig. 2:**
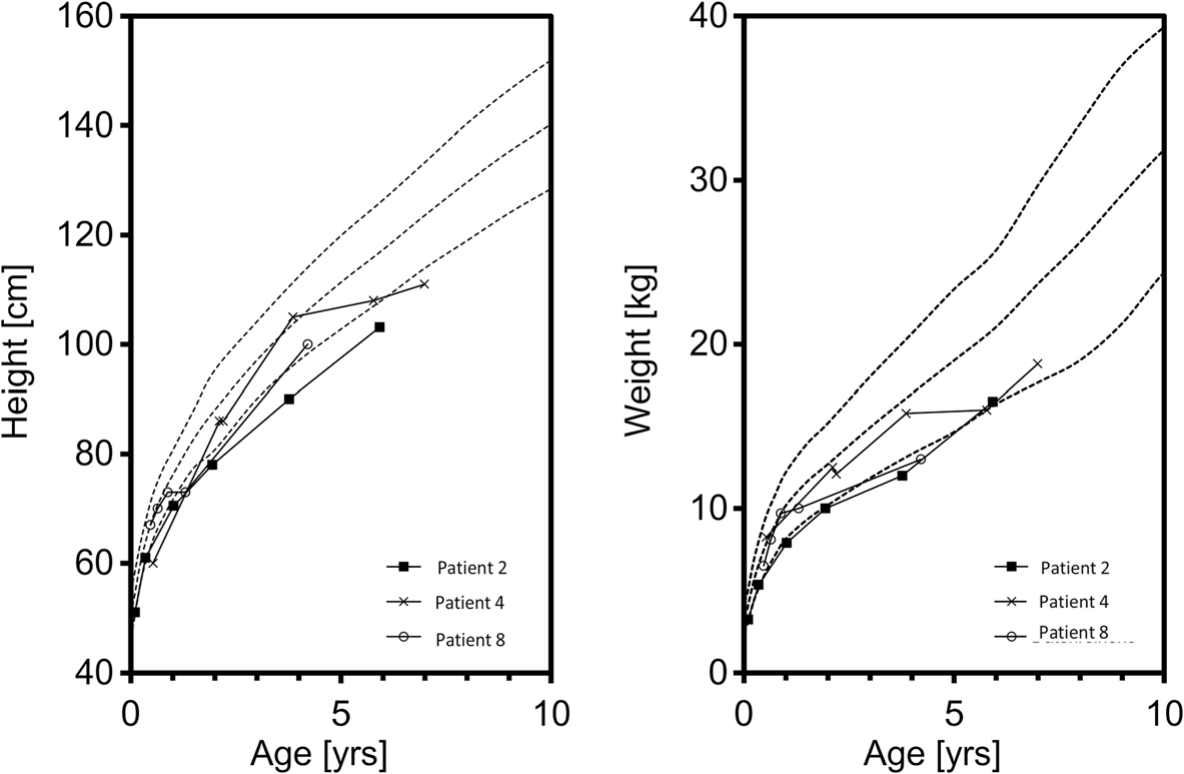
The 3rd, 50th, and 97th percentiles according to the height and weight references of Prader for male patients with a poor height and weight development. Patient 2 had both poor height and weight gain. Patient 4 presented a good weight gain but had a poor height development. Patient 8 showed poor weight development but had catch-up growth

Five patients had an adequate height and weight development parallel with the 50th according to the height and weight references of Prader [17]. The remaining six patients presented with insufficient height and weight development as shown in the Figs. 1 and 2, respectively.

## Discussion

Surgery is the only cure for TCA. The surgical treatment for TCA has two goals: to remove the aganglionic intestine and to provide patients with a good quality of life which is reflected in an acceptable frequency of bowel movements, fecal continence and no symptoms of enterocolitis. Loss of the entire colon lead to reduced fluid retention and accelerated passage of intestinal contents. As a consequence, the patients who have undergone surgical treatment for TCA present initially a high frequency of bowel movements which lead to perianal excoriation. Bischoff et al. reported that in their series, perianal dermatitis was the second most common complication in patients with TCA [6]. In our study, perianal dermatitis occurred in all patients. For managing perianal dermatitis after the pull-through procedure, we observed that our patients had a favourable response to the local application of zinc oxide based ointments, which helped the vast majority of them to overcome this problem after a reasonable period of time. To avoid this complication, Bischoff et al. suggested keeping the ileostomy open until the child is toilet trained for urine, and can sit on a potty. Following this concept, none of the patients treated by the group from Cincinnati suffered from perianal dermatitis postoperatively [6].

Fecal incontinence and soiling severely impact the quality of life in children with TCA and was reported by some authors in 25% of patients at long-term follow-up [12]. However, data regarding *functional outcomes* after surgical treatment for TCA is heterogenic. On one hand, there is a lack of a clear definition of fecal incontinence, which makes it difficult to compare study results [12]. On the other hand, the low TCA incidence precludes extensive personal experience and limits the number of series in which large cohorts are assessed [3, 12, 16, 18, 19]. Often, cases with additional small bowel aganglionosis of varying levels are included in these series [3, 16, 18], which may suggest more pessimistic functional outcomes and quality of life for patients with TCA. Although new surgical techniques have been employed, in which variable lengths of aganglionic colon have been anastomosed to the normal small bowel with the intention to increase the consistency of the stools, the rate of fecal incontinence has not shown significant improvement over time [13]. Moreover, these techniques, apart from being technically difficult, are associated with a high incidence of postoperative enterocolitis [20]. Some surgeons, however, have reported very good results after total colectomy with straight ileoanal anastomosis [5, 12, 21]. Using this technique, Dodero et al. encountered no enterocolitis, no mortality, and good patient quality of life [21]. Although patients undergoing a straight ileoanal anastomosis were reported to initially have increased bowel movement frequency, this problem tends to improve over time since the small intestine adapts by increasing water and sodium reabsorption in the terminal ileum [6, 22]. We also performed a straight ileoanal anastomosis in the majority of our patients. We chose to this approach because the definitive operation started as a transanal endorectal pull-through procedure and the extent of aganglionosis was established at the time of definitive surgery by intraoperative frozen sections. The results we obtained are encouraging and support the observations made by Dodero et al. [21], who performed a total colectomy with ileoanal anastomosis in his patients with TCA, as mentioned above. With the exception of the patient who eventually had a permanent ileostomy, all examined patients were continent. Stool frequency was high after surgery (10–15 stools/day) and led to excoriation of the perianal region in all patients. Bowel movement frequency decreased with time, reaching a median of 5 stools/day after a median follow-up of 78 months. All evaluated patients were continent and the survey revealed a high overall satisfaction in terms of functional results, with a median modified Wildhaber score of 14.5 points. Regarding quality of life, the scores for patients and parents were higher than expected, reaching a median score of 14/15 points. Although the majority of patients reported a good quality of life, this can result from adaptations to certain disease states in addition to development of alternative strategies by patients and parents that are aimed at preventing disease-associated functional failures. The use of these alternative strategies cannot be excluded in the present study.

Children with surgically repaired HD may also develop obstructive gastrointestinal symptoms and or enterocolitis due to a functional obstruction caused by an inability of the internal anal sphincter to relax [23]. Anal sphincter injections with BoTox may provide a reversible therapy for these patients. Chumpitazi et al., after analysing the long-term clinical outcomes of botulinum toxin therapy in children with non-relaxing internal anal sphincter observed a clinical improvement in 89% of the patients after the first injection. Approximately 77% of the patients required multiple injections. An excellent or good long-term outcome that was maintained for a mean of 17 months after the last injection was seen in 53% of the patients [23]. In our group, two patients had a non-relaxing internal anal sphincter after surgical treatment. An obvious clinical improvement was seen in both patients after anal sphincter injections with BoTox, although by one of them multiple injections were necessary.

Failure to thrive has been reported in patients who underwent total colectomy for TCA [14, 24]. The degree of failure to thrive, however, seems to be related to the extent of aganglionosis. Ikawa et al. reported on a statistically significant negative linear correlation between body weight and height and the extent of aganglionosis, in which patients with ileal involvement < 10 cm achieved a normal body weight development and height [14]. Our findings are in contrast with these observations. Although the level of aganglionosis did not exceeded more than 10 cm of terminal ileum, the majority of our patients had an inadequate growth and weight development. Therefore, a comprehensive follow-up, utilizing an interdisciplinary team of surgical, gastroenterological, and dietary specialists, might help to improve the weight and height development of these patients.

The prevalence of *anemia and iron deficiency* after colectomy for causes other than TCA has been reported to be between 5 and 56% in the adult population [25]. There are only a few reports that have addressed anemia after surgical treatment for TCA in children. Ikawa et al. reported on serum iron deficiency in children with TCA and ileal involvement > 10 cm [14]. Since almost all dietary iron is absorbed in the duodenum and the upper jejunum, iron deficiency in TCA patients is unlikely to be caused by the extent of ileal involvement, but rather from other reasons. These may include chronic occult bleeding from chronic nonspecific inflammatory processes in the remaining gut [14]. In our series, 50% of the examined patients had a low saturation of transferrin in serum, although all except one patient had normal hemoglobin levels. In this patient, a J-Pouch ileoanal anastomosis had been carried out, and endoscopic examination of the pouch revealed no signs of pouchitis. All examined patients had a negative hemoccult tests.

Patients who have undergone a total colectomy are also reported to develop *vitamin B12 deficiency* [25]. There are three possible causes for vitamin B12 deficiency in patients after total colectomy: 1.) reduced absorptive capacity because of resection of the last 16 to 60 cm of terminal ileum; 2.) bacterial overgrowth; or 3.) dietary intolerance. Vitamin B12 and folic acid serum levels were normal in all our patients, and none showed signs of malabsorption or bacterial overgrowth. In our population The ileal involvement did not exceeded 7 cm of the terminal ileum. None of our patients suffered from dietary intolerance.

Our study has certain limitations. An important limitation is represented by the retrospective nature of the study. Another limitation lies in the small number of the patients included in the study, which is justified by the rarity of these disease.

## Conclusion

In conclusion, TCA represent a disease which is difficult to diagnose, and once diagnosed it may pose pre- and postoperative management challenges. Although the majority of patients reported a good quality of life, this can result from adaptations to certain disease states in addition to development of alternative strategies by patients and parents that are aimed at preventing disease-associated functional failures. The failure to thrive seems to be related with the extent of aganglionosis. The inclusion of these patients in interdisciplinary long-term follow-up care, in which pediatric surgeons, gastroenterologists, and dieticians are involved, is essential.

## Additional files

Additional file 1: Classification of surgical complications after Dindo and Clavien. Ranking system proposed by Dindo and Clavien [15] for classification of postoperative complications. (DOCX 12 kb)

Additional file 2: Modified Wildhaber continence score. Modified scoring system from Wildhaber [16] to objectively assess the functional outcome (continence) of patients who underwent a pull-through procedure for HD. (DOCX 13 kb)

Additional file 3: Modified Barrena score. Modified scoring system from Barrena [12] to assess the quality of life after surgical treatment in patients with TCA. (DOCX 13 kb)

## Abbreviations

HD: Hirschsprung’s disease
TCA: Total colonic aganglionosis
