# Supplementary material for: Surgical treatment of children with total colonic aganglionosis: functional and metabolic long-term outcome
Source: BMC Surg. 2018 Aug 15;18:58. doi: 10.1186/s12893-018-0383-6 (PMC6094876; doi:10.1186/s12893-018-0383-6)
Supplement: Supplementary file 1 — Classification of surgical complications after Dindo and Clavien. Ranking system proposed by Dindo and Clavien [15] for classification of postoperative complications. (DOCX 12 kb) [file 12893_2018_383_MOESM1_ESM.docx]

**Classification of surgical complications after Dindo and Clavien**

| **Grade** | **Definiton** |
| --- | --- |
| **I** | Any deviation from the normal postoperative course without the need for pharmacological treatment or surgical, endoscopic, and radiological interventions  Allowed therapeutic regimens are: drugs as antiemetics, antipyretics, analgetics, diuretics, electrolytes, and physiotherapy. This grade also includes wound infections opened at the bedside |
| **II** | Requiring pharmacological treatment with drugs other than such allowed for grade I complications. Blood transfusions and total parenteral nutrition are also included |
| **IIIa** | Complication requiring surgical, endoscopic or radiological intervention without general anesthesia |
| **IIIb** | Complication requiring surgical, endoscopic or radiological intervention under general anesthesia |
| **IVa** | Life-threatening complication requiring ICU management by single organ dysfunction |
| **IVb** | Life-threatening complication requiring ICU management by multiple organ dysfunction |
| **V** | Death of the patient |
