# Supplementary material for: Surgical treatment of children with total colonic aganglionosis: functional and metabolic long-term outcome
Source: BMC Surg. 2018 Aug 15;18:58. doi: 10.1186/s12893-018-0383-6 (PMC6094876; doi:10.1186/s12893-018-0383-6)
Supplement: Supplementary file 2 — Modified Wildhaber continence score. Modified scoring system from Wildhaber [16] to objectively assess the functional outcome (continence) of patients who underwent a pull-through procedure for HD. (DOCX 13 kb) [file 12893_2018_383_MOESM2_ESM.docx]

**Modified Wildhaber continence score**

| **Items** | **Score: 2** | **Score: 1** | **Score: 0** |
| --- | --- | --- | --- |
| Stool frequency (day) | 1-2 × | 3-5 × | >5 × |
| Stool frequency (night) | 1-2 × | 3-5 × | >5 × |
| Stool consistency | normal | loose | liquid |
| Urge to defecate | normal | occasionally | seldom/absent |
| Soiling | none | occasionally | permanently |
| Difficulties to retain stool | none | occasionally | permanently |
| Discrimination normal/fluid stool | normal | deficient | absent |
| Diapers required | none | occasionally | permanently |
| Medications required | none | occasionally | Permanently |
| **Range of score** | **13-18** | **7-12** | **0-6** |
| **Functional outcome** | **Good** | **Fair** | **Poor** |
