# Supplementary material for: Surgical treatment of children with total colonic aganglionosis: functional and metabolic long-term outcome
Source: BMC Surg. 2018 Aug 15;18:58. doi: 10.1186/s12893-018-0383-6 (PMC6094876; doi:10.1186/s12893-018-0383-6)
Supplement: Supplementary file 3 — Modified Barrena score. Modified scoring system from Barrena [12] to assess the quality of life after surgical treatment in patients with TCA. (DOCX 13 kb) [file 12893_2018_383_MOESM3_ESM.docx]

**Barrena score regarding quality of life**

| **Items** | **Score:3** | **Score:2** | **Score:1** |
| --- | --- | --- | --- |
| Dietary habits | no restrictions | some restrictions | serious restrictions |
| School | normal activity | serious impairment of daily activities | no activities |
| Free time | no restrictions | limited restrictions | serious restrictions |
| Impact on personality | no impact | some impact | serious impact |
| Impact on family life | no impact | some impact | serious impact |
| **Range of score** | **11-15** | **6-10** | **0-5** |
| **Quality of life** | **Good** | **Fair** | **Poor** |
